# Combined analysis of cell growth and apoptosis-regulating proteins in HPVs associated anogenital tumors

**DOI:** 10.1186/1471-2407-10-118

**Published:** 2010-03-27

**Authors:** Tsuyoshi Mitsuishi, Yukie Iwabu, Kenzo Tokunaga, Tetsutaro Sata, Takehiko Kaneko, Kuniaki Ohara, Ikuroh Ohsawa, Fumino Oda, Yuko Yamada, Seiji Kawana, Kohji Ozaki, Mayuka Nakatake, Osamu Yamada

**Affiliations:** 1 Department of Dermatology, Nippon Medical School, 1-1-5 Sendagi, Bunkyou-ku, Tokyo, 113-8603, Japan; 2 Department of Pathology, National Institute of Infectious Diseases, Toyama 1-23-1, Shinjuku-ku, Tokyo 162-8640, Japan; 3 Department of Dermatology, The Fraternity Memorial Hospital, 2-1-11 Yokoami, Sumida-ku, Tokyo, 130-8587, Japan; 4 Department of Dermatology, Toranomon Hospital, 2-2-2 Toranomon, Minato-ku, Tokyo, 105-8470, Japan; 5 Department of Biochemistry and Cell Biology, Institute of Development and Aging Sciences, Graduate School of Medicine, Nippon Medical School, 1-396 Kosugi-cho, Nakahara-ku, Kawasaki City 211-8533, Japan; 6 Department of Hematology, Tokyo Women's Medical University, 8-1 Kawada-cho, Shinjyuku-ku, Tokyo, 162-8666, Japan

## Abstract

**Background:**

The clinical course of human papillomavirus (HPV) associated with Bowenoid papulosis and condyloma acuminatum of anogenital tumors are still unknown. Here we evaluated molecules that are relevant to cellular proliferation and regulation of apoptosis in HPV associated anogenital tumors.

**Methods:**

We investigated the levels of telomerase activity, and inhibitor of apoptosis proteins (IAPs) family (c-IAP1, c-IAP2, XIAP) and c-Myc mRNA expression levels in 20 specimens of Bowenoid papulosis and 36 specimens of condyloma acuminatum in anogenital areas. Overall, phosphorylated (p-) AKT, p-ribosomal protein S6 (S6) and p-4E-binding protein 1 (4EBP1) expression levels were examined by immunohistochemistry in anogenital tumors both with and without positive telomerase activity.

**Results:**

Positive telomerase activity was detected in 41.7% of Bowenoid papulosis and 27.3% of condyloma acuminatum compared to normal skin (*p *< 0.001). In contrast, the expression levels of Bowenoid papulosis indicated that c-IAP1, c-IAP2 and XIAP mRNA were significantly upregulated compared to those in both condyloma acuminatum samples (*p *< 0.001, *p *< 0.001, *p *= 0.022, respectively) and normal skin (*p *< 0.001, *p *= 0.002, *p *= 0.034, respectively). Overall, 30% of Bowenoid papulosis with high risk HPV strongly promoted IAPs family and c-Myc but condyloma acuminatum did not significantly activate those genes. Immunohistochemically, p-Akt and p-S6 expressions were associated with positive telomerase activity but not with p-4EBP1 expression.

**Conclusion:**

Combined analysis of the IAPs family, c-Myc mRNA expression, telomerase activity levels and p-Akt/p-S6 expressions may provide clinically relevant molecular markers in HPV associated anogenital tumors.

## Background

Telomerase is a ribonucleoprotein reverse transcriptase (RT) complex that adds telomeric TTAGGG repeat sequences to chromosomes by using an intrinsic RNA component as template [1,2]. Human telomerase consists of RNA components and a catalytic subunit of human telomerase RT (hTERT), and is coimmunoprecipitated with Akt kinase, heat shock protein 90, the mammalian target of rapamycin (mTOR), and p70 ribosomal protein S6 kinase 1 (S6K1) [3]. Transcription factors c-Myc, SP1, estrogen receptor, E2F-1, WT-1, NFκB and MZF-2 are involved in regulation of hTERT gene expression [4]. Telomerase activity (TA), an indicator of cellular immortalization, is considered to at least partly responsible for the unlimited replication seen in immortalized cells [1,5]. Previous studies have shown that malignant skin neoplasias, including melanoma, exhibit high levels of TA compared to benign skin conditions [6]. High levels of TA in such malignant cells may act to maintain stable telomere lengths [7].

In general, cutaneous tumors of the anogenital area are usually associated with human papillomaviruses (HPVs). High and low risk HPV types are usually detected in Bowenoid papulosis (BP) and condyloma acuminatum (CA), respectively. BP is a rare skin neoplasia, which is found in the anogenital area as multifocal papular lesions. In contrast, CA occasionally occurs as pigmented nodules or papules in the anogenital area, resulting sometimes in its misdiagnosis as BP [8]. Overall, high and low risk HPV types were sometimes found in BP [9,10]. Recently, E6 oncoproteins belonging to high risk HPV types were shown to increased TA in epithelial cells, predominantly by inducing transcription of the hTERT gene [11]. In BP and CA, some anogenital tumors show rapid growth and mitosis, while others show spontaneous remission. Therefore, it is clinically difficult to determine whether it is necessary to treat HPV associated anogenital tumors.

The phosphorylated (p-) proteins associated with cell signaling pathways, e.g., Akt, 4E-binding protein 1 (4EBP1) and ribosomal protein S6 (S6), provide proliferative signals, promote survival and regulate protein synthesis [12]. Among these proteins, protein kinase Akt is activated by phosphorylation of threonine 308, and serine 473 has been shown to be important in mediating cell proliferation and inhibiting apoptosis in several tissues and cancers [13,14]. The Akt activates many downstream factors, including mTOR, which in turn activates 4EBP1 and p70S6K1. The 4EBP1 and p70S6K1 play a critical role in controlling ribosomal protein synthesis during cell proliferation [15,16] through the phosphorylation of eukaryotic initiation factor 4E and S6, respectively. However, expression of p-AKT, p-S6 and p-4EBP1 in anogenital tumors has not been previously reported.

Recently, inhibitors of apoptosis protein (IAP) family members, e.g., c-IAP1, c-IAP2 and XIAP, were reported to act as tumor markers and were used as prognostic factors for estimating the survival of cancer patients. There is a large body of data demonstrating elevated expression of IAP proteins in many human cancers [17-19]. Among them, cervical cancer cells with high risk HPV types have been investigated for the expression of IAPs family [20,21], however, there are no reports about the expression of those genes of HPV associated anogenital tumors. The majority of HPV associated tumor cells possess active TA, and a combination of active TA and high risk HPV oncogene proteins E6 and E7 contributes to the immortalization of primary epithelial cells. The E6 of HPV directly interacts with c-Myc, and the complex activates hTERT expression [11]. Recently, the transcription of c-IAP2, a member of the IAP protein family, was shown to be significantly upregulated by E6 and E7 oncoproteins in cells infected with high risk type HPV 16, but not in cells infected with low risk type HPV 6. Among IAPs family members, c-IAP2 is related to hTERT protein and promotes hTERT as well as c-Myc activity in E6 of HPV 16 [22]. To evaluate molecules relevant to cellular proliferation and immortalization, we examined the expression of the IAPs family members and c-Myc in CA and BP by real time RT-PCR. In addition, we present the TA level and correlate a positive TA with the frequency of p-Akt, p-S6 and p-4EBP1 expression in these tumors.

## Methods

### Patients and tissues samples

A total of 74 samples (36 CA, 20 BP, and 18 normal skin (NS)) obtained from 74 Japanese patients were examined in the current study. Biopsy or surgical specimens were collected at the Department of Dermatology, Nippon Medical School, Japan. Initially, 12 BP and 22 CA specimens were collected and were immediately placed in liquid nitrogen and stored at - 80°C until proteins and DNAs extraction. A second portion of each samples was fixed in 10% buffered formalin solution and embedded in paraffin for histopathologic examination, and immunohistochemistry. Eight BP and 14 CA samples were collected to examine the expression of IAPs family and c-Myc by real time RT-PCR. In addition, initial remained 2 BP and 1 CA frozen samples were used for real time RT-PCR. Samples of NS were collected from a remainder of non sun-exposed skin, which was used as donor skin for performing a skin graft. None of the donor patients had a history of lymphatic disorders or exhibited active atopic dermatitis. Informed consent was obtained from each patient, and the study was approved by the Ethical Committee at Nippon Medical School and National Institute of Infectious Diseases.

### Preparation of tissue extracts

The frozen tissues consisting of 12 samples out of 20 BP, 22 samples out of 36 CA, and 14 samples out of 18 normal skin were added in ice lysis buffers and homogenized gently {(105 cells/20 μl) in ice-cold lysis buffer [10 mM tris-HCl (pH 7.5), 1 mM MgCl2 1 mM EDTA, 0.1 mM PMSF, 5 mM β-mercaptoethanol, 0.5% 3-[(3-Cholamidopropyl)dimethylammonio]-1-propane-sulfonate (CHAPS), and 10% glycerol]}. The suspension was incubated for 30 min on ice and then centrifuged at 100,000 g for 30 min at 4°C. The supernatant was removed and the protein concentration was determined by the Bradford assay (Bio-Rad, Hercules, CA).

### Nonradioisotope telomere repeat amplification protocol (TRAP) assay

Telomerase activity (TA) was assayed by the TRAP method in a total volume of 25 μl, as previously described [5,23,24]. In brief, aliquots of untreated cell extract were incubated with 0.1 μg of TS oligonucleotide primer (5'-AATCCGTCGAGCAGAGTT-3') for 20 min at 22°C in the following reaction mixture: 20 mM Tris-HCl (pH 8.3), 1.5 mM MgCl2, 63 mM KCl, 0.005% Tween-20, 1 mM EDTA, 50 μM dNTPs, 1 μg of T4 gene 32 protein (Roche, Mannheim, Germany), and 0.1 mg/ml BSA. Following elongation of the TS oligonucleotide primer by telomerase, the mixture was incubated at 95°C for 5 min prior to the addition of 5 units of *Taq *polymerase and 0.1 μg CX oligonucleotide primer (5'-CCCTTACCCTTACCCTTACCCTAA-3'). The elongated products were amplified by PCR (30 cycles at 94°C for 30 s, 50°C for 30 s, and 72°C for 60 s). One-fourth of each reaction product was then analyzed by electrophoresis in 1× Tris-glycine on 12.5% polyacrylamide nondenaturing gels and visualized with SYBR Green DNA stain (BMA Rockland, ME, USA). Internal telomerase assay standard (ITAS) was used as an internal control [25].

### Quantification of enzyme activity

Quantification of enzyme activity in samples was determined using NIH imaging software [25]. In brief, the signal intensity of each band on the TRAP ladders was measured individually. These values were compared with TRAP products obtained with known amounts of control extract, assayed in parallel. The relative specific TA of each sample is expressed as a percentage of the specific activity of the control extracts. An extract of EB virus transformed B cell lines (Namalva cells) was used as a positive control. We estimated that more than 20% of relative TA value mean having significant enzymatic activity in individual sample compared with Namalva cells and normal skin samples by calculating with NIH imaging soft ware.

### Real Time RT-PCR and gene expression analysis

Real time RT-PCR was performed in order to evaluate the differences in the expression of IAPs family members (c-IAP1, c-IAP2, X-IAP) and c-Myc between BP, CA and NS. The frozen tissues (10 of 20 BP samples, 15 of 36 CA samples, and 4 of 18 NS samples) were used for real time RT-PCR and gene expression analysis. The mRNA transcripts of the IAPs family members and c-Myc were quantified using the quantitative kinetic reverse transcription PCR methodology (Applied Biosystems, Foster City, CA) and Taqman chemistry (Roche Applied Science, Penzberg, Germany). All expression levels were normalised to the expression of the housekeeping gene, GAPDH. Total RNA was extracted from the skin samples using an RNAqueous Kit (Ambion, Austin, TX) and treated with TURBO DNA-*free *(Ambion, Austin, TX) according to the manufacturer's protocols. The prepared RNAs were subjected to real time PCR using an Mx3005P thermocycler (Strategene, San Diego, CA) and a QuantiTect Multiplex RT-PCR Kit (Qiagen, Valencia, CA) [26]. Plasmids were assayed along with the tissue RNAs and used to construct standard curves. The specific probes and primers that were used are shown in Table 1.

**Table 1:** Sequences of specific probes and primers for real time RT-PCR

Gene	Application	Sequence	Length (bp)	5'	3'	Tm (°)	Genebank accession number
**GAPDH**	P	AAC AGC GAC ACC CAC TCC TCC ACC	24	FAM	BHQ1	67.4	NM_002046
		
	F	ACC AGG TGG TCT CCT CTG AC	20			58.5	
		
	R	TGT AGC CAA ATT CGT TGT CAT ACC	24			58.6	

**c-IAP1**	P	AGG AAA TGC TGC GGC CAA CAT CTT CA	26	ROX	BHQ2	66.8	NM_001166
		
	F	GAG AGA ACT GAT TGA TAC CAT TTT GG	26			57.6	
		
	R	AGA CCT GAA ACA TCT TCT GTT GG	23			57.3	

**c-IAP2**	P	TCC GTC AAG TTC AAG CCA GTT ACC CTC A	28	Cy5	BHQ3	67.2	NM_001165
		
	F	CTT GAT AAG AAT TAA AGG ACA GGA GTT C	28			58.0	
		
	R	TGG GCT GTC TGA TGT GGA TAG	21			57.5	

**XIAP**	P	CAG TGA AGA CCC TTG GGA ACA ACA TGC T	28	HEX	BHQ1	66.7	NM_001167
		
	F	GGA GGA GGG CTA ACT GAT TGG	21			58.1	
		
	R	TAT TCT TGT CCC TTC TGT TCT AAC AG	26			57.9	

**c-Myc**	P	CCA CCA CCA GCA GCG ACT CTG AGG A	25	HEX	BHQ1	69.0	NM_002467
		
	F	GGT GCT CCA TGA GGA GAC AC	20			58.1	
		
	R	CCA GCA GAA GGT GAT CCA GAC	21			58.7	

P; Probe, F; Forward, R; Reverse

### Immunohistochemistry

A total of 22 CA (5 with and 17 without TA) samples and 12 BP (5 with and 7 without TA) samples were analysed for telomerase activity were also examined immunohistochemically in order to compare the expression of p-Akt thr308, p-S6 and p-4E binding protein 1(4EBP1). Rabbit polyclonal antibodies against p-Akt thr308 (Santa Cruz Biotechnology, Santa Cruz, CA, #sc-16646-R, dilution1:400), p-S6 ser235/236 (Cell Signaling Technology, Danvers, MA, #2211, 1:200), and p-4EBP1 thr70 (Cell Signaling Technology, #9455, 1:50) were used. Immunohistochemistry was performed on the tissue sections from tumours with high or low TA that were mounted on aminopropyltriethoxysilane-treated slides (Matsunami Glass Ind., LTD., Osaka, Japan), as previously described [27].

### Immunohistochemical evaluation

The stained sections were independently examined by two of the authors (F.O. and Y. Y.). The labelling index of p-Akt thr308, p-S6, and p-4EBP1 was determined by assessing the number of stained cells within the relevant area of a selected section containing more than 50 intraepidermal cells, and at least 4 random microscopic fields at 200× magnification were evaluated. After counting both immunoreactive cells and the total number of intraepidermal cells, the average percentage of immunoreactive cells was calculated without knowledge of the clinical data. The percentage of positive cells was arbitrarily scored as follows: 0, <30%; 1, 30% to 60%; 2, >60% [28].

### Western blot

Western blot analysis and reverselysate protein rays were performed in anogenital tumors samples. Proteins were resolved on a polyacrylamide gel and transferred to a polyvinylidene difluoride (PVDF) membrane (Bio-Rad, Hercules, CA). The membrane was incubated with the primary antibodies and then the HRP-conjugated anti-rabbit IgG antibody. Immunoreactive bands were visualised with the ECL Plus Detection Reagent according to the manufacturer's protocol (GE Healthcare Bio-Sciences, Piscataway, NJ).

### Detection of HPV DNA and typing

Available frozen tissues from 10 BP and 10 CA samples were digested with proteinase K (100 mg/ml) in the presence of 0.5% SDS for 2 h at 65°C, and extracted with phenol-chloroform-isoamyl alcohol. The DNAs were then precipitatedin ethanol as previously described [29]. Total cellular DNA was used for PCR amplification using L1C1/L1C2(C2 m) primers [30]. The primer sets are able to amplify majority of mucosal HPV types including types 6 and 16. The HPV type was determined by comparing the restriction fragment length polymorphism (RFLP) pattern of the amplified sequences with those deduced from sequences of known HPV types in Genbank or EMBL Databank [30].

### Statistical methods

The differences between the cases were assessed using χ^2 ^analyses to compare categorical variables and the Mann-Whitney *U *test to compare continuous measures. Statistical analysis was carried out using the SPSS software package (SPSS Inc., version 17.0, Chicago, IL, USA). A two-sided test with a significance level of 0.05 was used to determine statistical significance for all analyses.

## Results

### Histopathologic examination

Histopathologic examination of 20 BP showed the presence of several dyskeratotic keratinocytes in a thickened epidermis with hyperchromatic nuclei. There were no significant histopathologic differences in 20 BP. In contrast, moderate to considerable acanthosis, with thickening and elongation of the rete ridges, contained many vacuolated cells of the stratum malpihii in 36 CA samples. These CA samples did not show typical Bowenoid changes. The anogenital tumors had equivalent numbers of keratinocytes and/or dyskeratotic keratinocytes.

### Telomerase activity

**Figure 1:**
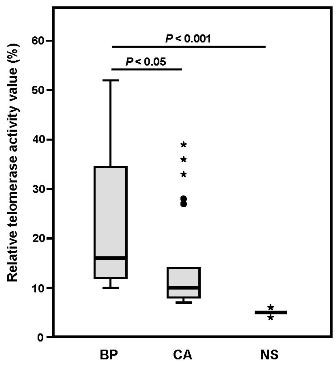
**The analysis of telomerase activity (TA) in the samples of Bowenoid papulosis, condyloma acuminatum, and normal skin by a nonisotopic PCR-based TRAP assy**. The box plot of relative telomerase activity among Bowenoid papulosis, condyloma acuminatum, and normal skin. Box plots show median, 25th and 75th percentiles and range. The extreme values were noted as dots and stars. Bowenoid papulosis samples had a significantly higher relative levels of TA than those with condyloma acuminatum and normal skin samples (condyloma acuminatum; *p *= 0.025, normal skin; *p *< 0.001, Mann-Whitney *U *test, two-sided).

Epstein-Barr (EB) virus transformed B cell lines (Namalva cells) and normal skin were used as positive and negative control, respectively. All the normal control skin samples exhibited very low or absent TA when using the same amount of protein (1 μg) for telomerase assay. In contrast, Namalva cells showed high TA and regarded as a 100% relative TA value. Moderate to high TA levels were visualized in 41.7% (5 out of 12) BP samples when compared to CA (*p *= 0.025) and normal non sun-exposed skin (*p *< 0.001). In contrast, 22.7% (5 out of 22) CA samples showed positive TA compared to normal non sun-exposed skin (*p *< 0.001) (Figure 1). Among anogenital tumors, high TA levels corresponding to greater than 40% of relative TA were detected in 25% (3 out of 12) BP samples, but none of the 22 CA samples.

### Detection of low risk or high risk of HPV DNA

Available10 BP and 10 CA samples were examined to determine the presence of HPV DNA by performing the PCR assay. Low risk type HPV 6 was detected in all of the CA samples, while high risk types HPV 16 and HPV 31 were detected in 9 BP samples and 1 BP sample, respectively.

### C-IAP1, C-IAP2 and XIAP mRNA expression

**Figure 2:**
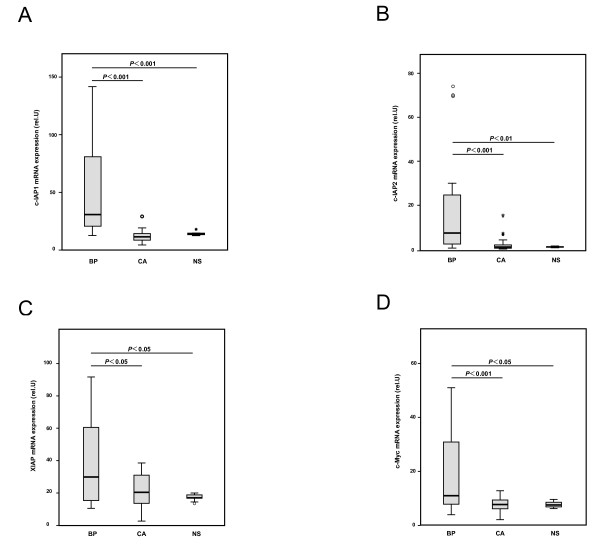
**Quantitative real time RT-PCR analysis of (A) c-IAP1 (B) c-IAP2, (C) XIAP and (D) c-Myc in Bowenoid papulosis, condyloma acuminatum, and normal skin**. The relative expression was calculated as the expression of the IAPs or c-Myc normalised to the expression of GAPDH. Each experiment was performed three times, and data are shown as box and whisker plots. The expression of (**A**) c-IAP1, (**B**) c-IAP2, (**C**) XIAP and (**D**) c-Myc was significantly higher in Bowenoid papulosis compared with condyloma acuminatum and normal skin. (**A**; condyloma acuminatum; *p *< 0.001, normal skin; *p *< 0.001 **B**; condyloma acuminatum; *p *< 0.001, normal skin; *p *= 0.002 **C**; condyloma acuminatum; *p *= 0.022, normal skin; *p *= 0.034 **D**; condyloma acuminatum; *p *< 0.001, normal skin; *p *= 0.01, Mann-Whitney *U *test, two-sided). In contrast, no significant difference in the expression of the IAP family members or c-Myc was detected between condyloma acuminatum and normal skin.

c-IAP1, c-IAP2, and XIAP were significantly upregulated in the BP samples compared to 15 CA samples (c-IAP1, *p *< 0.001; c-IAP2, *p *< 0.001; XIAP, *p *= 0.022; Figure 2A, B, C). Similarly, c-IAP1, c-IAP2, and XIAP mRNA levels in BP samples were significantly up-regulated compared to NS (c-IAP1, *p *< 0.001; c-IAP2, *p *= 0.002; XIAP, *p *= 0.034). To estimate the mRNA expression of the IAPs family, each BP sample was carefully examined in triplicate. Three of 10 BP cases (Cases 6, 7 and 10) showed a higher expression level of the IAP family members compared to other samples (vs. other BP (*n *= 7); c-IAP1, *p *< 0.001; c-IAP2, *p *< 0.001; XIAP, *p *< 0.001; and vs. CA (*n *= 15); c-IAP1, *p *< 0.001; c-IAP2, *p *< 0.001; XIAP; *p *< 0.001).

Although more than 27.3% of CA samples demonstrated positive TA, c-IAP1, c-IAP2, and XIAP expression levels were not significantly up-regulated when compared to the high expression levels observed in BP. This results suggested that the oncogenic proteins expressed by low-risk HPV types did not activate c-IAP1, c-IAP2 or XIAP. The expression levels of c-IAP1, c-IAP2 and XIAP in CA were similar to the levels seen in NS (c-IAP1, *p *= 0.052; c-IAP2, *p *= 0.876; XIAP, *p *= 0.098). Two of 15 CA samples (Cases 12 and 13) demonstrated an increased expression of only XIAP compared to NS samples, but this expression was a rare event in other CA samples.

### C-Myc mRNA expression

c-Myc mRNA expression showed significant differences between BP and CA samples (*p *< 0.001; Figure 2D). Similar to above, all measurements were performed in triplicate. Of 10 BP and 15 CA samples examined, 3 BP cases (Cases 6, 7 and 10) had significantly higher c-Myc expression compared to other samples (vs. other BP (*n *= 7); c-Myc, *p *< 0.001; and vs. CA (*n *= 15); c-Myc, *p *< 0.001). These three cases also showed higher expression levels of c-IAP1, c-IAP2 and XIAP mRNA, suggesting the potential for a high level of malignancy. These results are consistent with those of a previous study, which suggested that HPV E6 directly interacts with c-Myc and that c-Myc/E6 complex activates hTERT expression [11]. Similar to the levels of c-IAP1, c-IAP2 and XIAP, mRNA expression level of c-Myc was elevated in BP compared to NS samples (*p *= 0.010). In contrast, c-Myc expression in CA was similar to that of NS (*p *= 1.000).

### P-Akt 308, P-S6 and P-4EBP-1 expression

**Figure 3:**
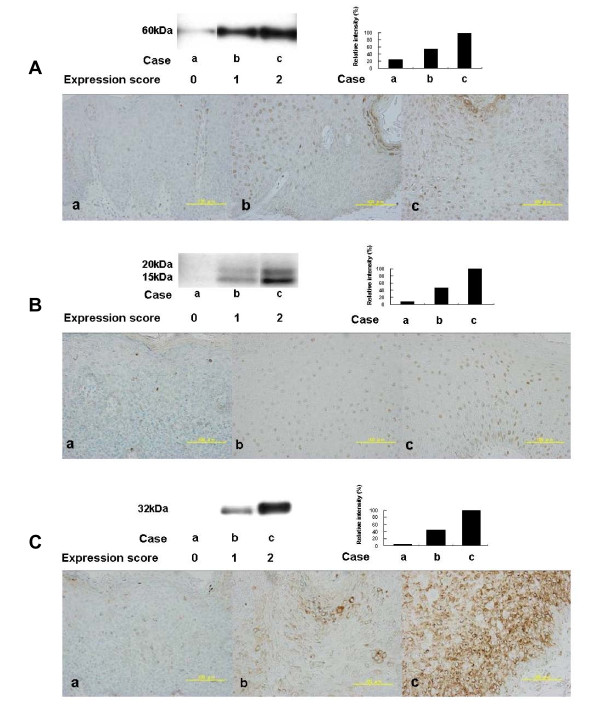
**Western blot analysis and immunohistochemical analysis for P-Akt thr308 (A), P-4EBP1 (B) and P-S6 (C) in Bowenoid papulosis and condyloma acuminatum**. Western blot validation of immunohistochemical stainings showed a good correlation. Cases b and c which exhibited more than 30% expressions of p-Akt thr308 and p-S6, showed the intensity bands using frozen samples. In contrast, more than 30% expression of p-4EBP1 was detected, with double bands at 15 and 20 kDa in Cases b and c. The expression score indicates 0, >30%; 1, 30% to 60%; 2, >60%. Scale bars = 100 μm.

**Figure 4:**
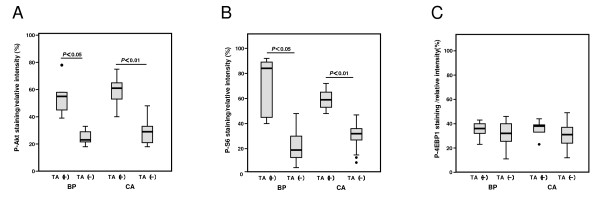
**P-Akt thr308, P-S6 and P-4EBP1 expressions in relation with telomerase activity**. Box graphs comparing positive telomerase activity (TA) for (A) P-Akt thr308, (B) P-S6 and (C) P-4EBP1 expressions in Bowenoid papulosis with and without TA (**A**; *p *= 0.004, **B**; p = 0.012, **C**; *p *= 0.807, Mann-Whitney *U *test, two-sided) and in condyloma acuminatum with and without TA (**A**; *p *= 0.001, **B**; *p *= 0.001, **C**; *p *= 0.307, Mann-Whitney *U *test, two-sided).

p-Akt expression was observed in the cytoplasm and nucleus of the intraepidermal cells (Figure 3A), while p-4EBP1 expression was mainly observed in the nucleus of intraepidermal cells (Figure 3B) and p-S6 expression was predominantly observed in the cytoplasm or at the edge of the nucleus (Figure 3C). The expression percentage was based on the estimated number of intraepidermal cells in each tissue section with positive staining, either in the nucleus or cytoplasm. Of 34 cases examined, BP and CA specimens with TA clearly demonstrated an increased incidence of both p-Akt expression (Figure 4A) and p-S6 expression (Figure 4B) compared to those samples without TA. In contrast, p-4EBP1 expression was not different between the anogenital tumors with or without TA (Figure 4C). Non sun-exposed normal skin demonstrated weak and occasionally moderate staining that was primarily localized to the basal layer (data not shown). In summary, there are differences in p-Akt308 and p-S6 expression, but not p-4EBP1 expression, between anogenital tumors with and without TA.

## Discusssion

Although a broad range of malignancies was investigated, telomerase activity (TA) of a comparative study has not been reported in Bowenoid papulosis (BP) and condyloma acuminatum (CA) of anogenital tumors. Despite of the infection with high risk HPV, BP sometimes shows spontaneous regressions within a few months. However, BP may transform into squamous cell carcinoma (SCC) of the genitalia but the mechanism for the development of SCC from BP has not been defined [8]. In contrast, CA is also infected with HPV which is low risk HPV types 6 or 11. Overall, giant CA is occationally considered a low grade of SCC [8]. The BP and CA are little known regarding the molecular mechanisms involved in malignant transformation or spontaneous remission based on the biological alterations of the cell cycle, cell signaling and apoptosis.

In order to identify if HPV is associated with TA in BP and CA of anogenital tumors, samples were tested by TRAP assay. The typical ladder pattern was detected. Positive TA was detected in 41.7% (5 of 12) BP samples and 22.7% (5 of 22) CA samples (*p *= 0.025). These results suggest that higher telomerase activity is correlated with high risk HPV associated BP compared to low risk HPV associated CA. There was no evidence of increased TA in normal skin (NS) from non-sun-exposed areas. Initially, we hypothesised that increased TA could be an important tumor marker for malignant potential. As expected, this study showed a higher frequency of increased TA in BP compared with CA. Importantly, increased TA was also found in 22.7% (5 out of 22) CA samples that were associated with low-risk HPV. In general, low levels of TA are present in non-malignant skin conditions [6]. Hiyama et al. demonstrated that leukocytes constitutively express low levels of TA [31]. Therefore, this study suggested that CA with TA may be an intermittent phenomenon and that the presence of low risk HPVs was not associated with increased TA. Overall, current study suggested that only the high risk HPV E6 and E7 genes increased TA in anogenital tumors.

The expression of the c-Myc gene is important for cell proliferation in HPV associated cutaneous lesions. The c-Myc gene acts directly or indirectly through its interaction with p53 and E6 oncoproteins, and plays an important role for the induction of hTERT protein [9]. In contrast, IAPs family (XIAP, c-IAP1 and c-IAP2) can suppress apoptosis induced by a variety of apoptotic triggers. The IAPs family can inhibit the downstream components of caspase activation pathways that regulate apoptosis. Some studies have established a circumstantial association between the IAPs family and cancer. The overexpression of several family members has been detected in several classes of human cancers [32,33]. Among IAPs family, c-IAP2 is related to the hTERT protein and promotes hTERT expression [22]. According to one study, overexpression of E6/E7 was found in high risk types HPV 16 and 18, but not in low risk type HPV 6, and an activated c-IAP2 promoter was found in E6/E7-immortalized human keratinocytes [22]. We quantitated the expression of IAPs family and c-Myc mRNA using real time RT-PCR assay in 10 BP and 15 CA samples. The levels of IAPs family members (c-IAP1, c-IAP2 and XIAP) and c-Myc mRNA expression in BP samples were higher than those in CA and normal skin samples (Figure 2). The mRNA expression levels were carefully determined by performing triplicate assays. Of interest, 30% (Cases 6, 7 and 10) of BP showed significantly high expression of c-IAP1, c-IAP2 and XIAP genes compared to the other samples. Increased c-Myc expression was also found in 30% (Cases 6, 7, and 10) of BP, and was not observed in CA samples. These results supported that some BP with high risk HPV types have high levels of antiapoptosis, while CA does not have such characteristics. In Cases 6, 7 and 10, it would be necessary to remove the lesional skin, because BP may undergo transformation into SCC. In contrast, the low expression of mRNA IAPs family members in other BP and CA samples suggested limited cell proliferation and a possible tendency toward spontaneous remission.

The phophatidylinositol-3 kinase (PI3K)/Akt signaling pathway plays a central role in diverse cellular functions, including proliferation, growth, survival and metabolism. In general, Akt promotes protein synthesis and cell growth by alleviating TSC1/2 suppression of the mammalian target of rapamycin (mTOR), allowing the latter to act as part of the mTOR-raptor complex on 4E-binding protein 1 (4EBP1) and ribosomal protein S6 (S6) [34]. Both S6 and p70S6 kinase1 are present downstream in the Akt signaling pathway, which is not linearly associated with 4EBP1 phosphorylation [35]. To date, alterations of the Akt/mTOR pathway have been observed in numerous types of carcinomas, but little has been reported about Akt/mTOR signaling in skin tumors. In the current study, although p-4EBP1 expression did not differ between CA and BP with or without positive TA, p-Akt and p-S6 were expressed significantly more often in BP and CA with increased TA than in CA and BP without TA. This result suggested that positive TA is associated with activated p-Akt and p-S6 expression but not with p-4EBP1 expression. Overall, we speculated that HPV associated anogenital tumor cells with increased TA could be correlated with activation of Akt/mTOR/p70S6K1/S6 signaling pathway but not with 4EBP1 signaling pathway. In fact, S6 and 4EBP1 are located in the downstream of Akt but they are different signal pathways [34,35].

## Conclusion

Our study showed there were differences between TA levels in BP and CA of anogenital tumors. A minority of BP samples with high risk HPV types showed increased c-Myc and IAPs family mRNA expression, whereas CA samples with low risk HPV types showed little increase in those genes. These results provided the majority of CA even if they have increased TA and/or positive proteins expression in cell signaling pathway, this may be transient or intermittent phenomenon and may be indicative of future apoptosis. The majority of BP lesions in this study showed low levels of TA and mRNA expression of IAPs family and c-Myc, suggesting most of BP were considered to be less aggressive tumors. However, BP with high TA levels and increased mRNA expression of IAPs family and c-Myc may develop into SCC. Therefore, overexpression of IAPs family and c-Myc are also important in affecting cancer progression. Combined estimation of cell growth and apoptosis-regulating proteins may provide clinically relevant molecular markers in HPV associated anogenital tumors.

## Abbreviations

TA: telomerase activity; HPVs: human papillomaviruses; c-IAPs: cellular inhibitor of apoptosis proteins; real time RT-PCR: real time reverse transcription polymerase chain reaction; p-Akt: phosphorylated Akt; S6: ribosomal protein S6; 4EBP1: 4E-binding protein 1; SCC: squamous cell carcinoma; BP: Bowenoid papulosis; CA: condyloma acuminatum; NS: normal skin.

## Competing interests

The authors declare that they have no competing interests.

## Authors' contributions

TM, TK, KO, and SK collected and analyzed clinical and pathological data, including patients' follow up. FO, and YY performed laboratory work and helped with data analysis. TM, KT, YI, TS, IO, KO, MN, and OY performed laboratory work, evaluated gene and protein expression, immunohistochemistry, and statistical analysis. TM, and OY wrote the manuscript. All the authors revised and approved the final version.

## Pre-publication history

The pre-publication history for this paper can be accessed here:

http://www.biomedcentral.com/1471-2407/10/118/prepub
